# Impact of Genetic Variation in *SORCS1* on Memory Retention

**DOI:** 10.1371/journal.pone.0024588

**Published:** 2011-10-26

**Authors:** Christiane Reitz, Joseph H. Lee, Robert S. Rogers, Richard Mayeux

**Affiliations:** 1 Taub Institute for Research on Alzheimer's Disease and the Aging Brain, College of Physicians and Surgeons, Columbia University, New York, New York, United States of America; 2 Gertrude H. Sergievsky Center, College of Physicians and Surgeons, Columbia University, New York, New York, United States of America; 3 Department of Neurology, College of Physicians and Surgeons, Columbia University, New York, New York, United States of America; 4 Department of Psychiatry, College of Physicians and Surgeons, Columbia University, New York, New York, United States of America; 5 Department of Medicine, College of Physicians and Surgeons, Columbia University, New York, New York, United States of America; 6 Department of Epidemiology, School of Public Health, Columbia University, New York, New York, United States of America; Boston University School of Medicine, United States of America

## Abstract

**Objective:**

We previously reported that genetic variants in *SORCS1* increase the risk of AD, that over-expression of SorCS1 reduces γ-secretase activity and Aβ levels, and that SorCS1 suppression increases γ-secretase processing of APP and Aβ levels. We now explored the effect of variation in *SORCS1* on memory.

**Methods:**

We explored associations between *SORCS1*-SNPs and memory retention in the NIA-LOAD case control dataset (162 cases,670 controls) and a cohort of Caribbean Hispanics (549 cases,544 controls) using single marker and haplotype analyses.

**Results:**

Three SNPs in intron 1, were associated with memory retention in the NIA-LOAD dataset or the Caribbean Hispanic dataset (rs10884402(A allele:β = −0.15,p = 0.008), rs7078098(C allele:β = 0.18,p = 0.007) and rs950809(C allele:β = 0.17,p = 0.008)) and all three SNPs were significant in a meta-analysis of both datasets (0.002<p<0.03). The corresponding A-T-T haplotype for these SNPs was associated with lower scores in both datasets (p = 0.02,p = 0.0009), and the complementary G-C-C haplotype was associated with higher scores in NIA-LOAD (p = 0.02). These associations were restricted to cases.

**Conclusions:**

Variation in intron 1 in *SORCS1* is associated with memory changes in AD.

## Introduction

The putative culprit in Alzheimer's disease (AD) is the amyloid β (Aβ) protein. It is produced by β-secretase (BACE) cleavage of the amyloid precursor protein (APP) at the N-terminus of the Aβ peptide followed by γ-secretase cleavage of the membrane-bound C-terminal APP fragment [1]. APP and the secretases are integral transmembrane proteins, and are dynamically sorted into the plasma membrane and the membranes of intracellular organelles [2], [3]. As a consequence, sorting mechanisms that cause APP and the secretases to colocalize in the same cellular compartment are expected to play important roles in the regulation of Aβ production.

We and several other groups have recently reported [4], [5], [6] that variants in the sortilin-related VPS10 domain containing receptor 1 (*SORCS1*), which maps to chromosome 10q_23–25_, are associated with AD. We also demonstrated that over expression of SorCS1 reduces γ-secretase activity and Aβ levels, and that suppression of SorCS1 increases γ-secretase processing of APP and the levels of Aβ. *SORCS1* belongs to the mammalian Vps10p-domain sorting receptor family, which is a group of five type I membrane homologues (SORL1, Sortilin, SorCS1, SorCS2, and SorCS3) [7], [8], [9], [10]. The common characteristic of these receptors is an N-terminal Vps10p domain, which either represents the only module of the luminal/extracellular moiety or is combined with additional domains. The individual receptors bind and internalize a variety of ligands, such as neuropeptides and trophic factors, and Sortilin and SorLA mediate trans-Golgi network-to-endosome sorting. Their prominent neuronal expression, several of the identified ligands, and recent results support the notion that members of this receptor family have important functions in neurogenesis, plasticity-related processes, and neuronal activity [11], [12] but their precise function remains elusive.

Based on these findings we hypothesized that genetic variants in the 5′ end in *SORCS1* might be associated with changes in memory performance, the cognitive domain predominately affected in AD. The goal of the present study was to investigate whether or not genetic variation in *SORCS1* was associated with memory retention in two independent datasets that have sufficient power to detect modest effect sizes.

## Methods

### Participants

Written informed consent was obtained from all subjects included. Recruitment for the Caribbean Hispanic Study was approved by the Institutional Review Board of the Columbia University Medical Center. Recruitment for the NIALOAD Study was approved by the relevant institutional review boards of the participating centers (ie. the IRBs of Boston University, Columbia University, Duke University, Indiana University, Massachusetts General Hospital, Mayo Clinic, Mount Sinai School of Medicine, Oregon Health & Science University, Rush University Medical Center, University of Alabama at Birmingham, University of California Los Angeles; University of Kentucky; University of Pennsylvania; University of Pittsburgh; University of Southern California; University of Texas Southwestern; University of Washington; Washington University Medical Center; University of Miami; Northwestern University; Emory University).The study was conducted according to the principles expressed in the Declaration of Helsinki.

The two datasets included a) 162 Caucasian cases and 670 controls from the NIA-LOAD study [13] and b) 549 cases and 544 controls from a Caribbean Hispanic dataset that have been described in detail elsewhere [14]. The clinical characteristics of these datasets are summarized in Table 1. The diagnoses of ‘probable’ or ‘possible’ AD were defined according to the National Institute of Neurological and Communication Disorders and Stroke–Alzheimer's Disease and Related Disorders Association (NINCDS-ADRDA) diagnosis criteria at clinics specializing in memory disorders or in clinical investigations. Persons were classified as “controls” when they were without cognitive impairment or dementia at last visit. Informed consent was obtained from all participants using procedures approved by institutional review boards at each of the clinical research centers collecting human subjects.

**Table 1:** Characteristics of the study samples.

	Caribbean Hispanic Study(n = 1,093)	NIA-LOAD Case Control(n = 832)
**Characteristics**		
Affected with AD	549	162
Unaffected	544	670
Age		
Onset: affecteds	79.98±8.0	71.6±6.9
Age at last exam: unaffecteds	78.87±6.4	76.1±8.4
Sex		
Proportion of females (%)	69.7	62.3
APOE allele frequency (%)		
e4	18.2	31.2
e3	75.1	63.3
e2	6.8	5.5

### Cognitive assessments

For both studies, all participants underwent a standardized neuropsychological test battery that examined multiple domains [15]. In the Caribbean Hispanic Study, orientation was evaluated using parts of the modified Mini-Mental State Examination [16]. Language was assessed using the Boston Naming Test [17], the Controlled Word Association Test [18], category naming, and the Complex Ideational Material and Phrase Repetition subtests from the Boston Diagnostic Aphasia Evaluation [19]. Abstract Reasoning was evaluated using WAIS-R Similarities subtest [20], and the non-verbal Identities and Oddities subtest of the Mattis Dementia Rating Scale [21]. Visuospatial ability was examined using the Rosen Drawing Test [22], and a matching version of the Benton Visual Retention Test [23]. Memory was evaluated using the multiple choice version of the Benton Visual Retention Test [23] and the seven subtests of the Selective Reminding Test [24]: total recall, long-term recall, long-term storage, continuous long-term storage, words recalled on last trial, delayed recall, and delayed recognition. This neuropsychological test battery has established norms for the same community [25]. In the NIA-LOAD Study, cognition was measured with a battery of 7 brief tests [26]. Working memory was assessed with Digit Span Forward [27], Digit Span Backward [27], and Digit Ordering [28]. Two measures of episodic memory were included: immediate and delayed recall of story A from the Wechsler Memory Scale-Revised [27]. Semantic memory was assessed by asking persons to name members of two semantic categories (Animals, Vegetables) in separate 1-min trials [26], [28], [29]. While all subjects recruited into the NIA-LOAD study underwent standard neuropsychological assessment that contributed to the diagnosis of AD, the standardized neuropsychological test battery especially designed for the NIA-LOAD study was integrated in the study at a later stage (year 2004). Therefore only 832 subjects of the originally recruited case-control sample (n = 1877) have standardized neuropsychological data and contributed to the final analytic NIA-LOAD sample included in the present analysis.

### Genotyping

Both study sites provided the results from genotyping of *SORCS1* SNPs that were part of its genome-wide studies described previously [13], [14]. For the NIA-LOAD study, SNPs were genotyped using the Illumina Human610Quadv1_B BeadChips (Illumina, San Diego, CA, USA). For the Caribbean Hispanic study, SNPs were genotyped using the Illumina HumanHap 650Y chip. Genotyping of *APOE* polymorphisms (based on SNPs rs7412 and rs429358) for all samples was performed at PreventionGenetics.

### Statistical methods

First, for both datasets a memory savings score was calculated by dividing Delayed Free Recall by Trial 6 Recall (Caribbean Hispanic Study) or by Story (NIA-LOAD Study) multiplied by 100 and expressed as a percent. Using the means and standard deviations from the control samples, we then transformed the resulting savings scores into z-scores. Then, we restricted the genotyping data to the SNPs that were overlapping in both datasets (110 overlappimg SNPs spanning 590 kb). SNP marker data were assessed for deviations from Hardy-Weinberg equilibrium (HWE) in controls. Independently for each dataset, multivariate linear regression analyses were used to assess genotypic and allelic associations with the memory savings scores, adjusting for Population stratification, sex, APOE-ε4 and age-at-onset or age-at-examination. The False Discovery Rate (FDR) [30], which controls the expected proportion of incorrectly rejected null hypotheses (type I errors), was used to account for the error in multiple comparisons. PLINK (http://pngu.mgh.harvard.edu/~purcell/plink/) was used to perform a meta-analysis of both datasets.

We used Haploview (http://www.broad.mit.edu/mpg/haploview/index.php) to assess linkage disequilibrium (LD). Haplotype blocks were defined using the confidence intervals algorithm. The default settings were used in these analyses, which create 95% confidence bounds on D′ to define SNP pairs in strong LD. Analyses assessing associations between haplotypes and the memory savings score, were carried out using a window of three contiguous SNPs using PLINK v1.07 (http://pngu.mgh.harvard.edu/~purcell/plink/) for case-control data. We performed all analyses first for cases and controls combined, and then for the case and control groups separately.

We also performed a meta-analysis of both datasets for single marker and haplotype analyses. To determine the strength of associations between the individual *SORCS1* SNPs (or haplotypes) and the memory savings score, we calculated a pooled OR for each marker/haplotype using fixed and random effects models using PLINK. We first performed meta-analyses of unadjusted results from the individual datasets, and then repeated the meta-analyses using the results from the individual datasets adjusting for Population stratification, sex, APOE-ε4 and age-at-onset or age-at-examination. The p-values for each SNP/haplotype were corrected for multiple testing using the False Discovery Rate (FDR). Between-dataset heterogeneity was quantified using the I^2^ metric for inconsistency and its statistical significance was tested with the chi-square distributed Q statistic. I^2^ is provided by the ratio of (Q−df)/Q, where df = the number of degrees of freedom (one less than the number of combined datasets); it is considered large for values above 50% and Q is considered statistically significant for p = 0.10.

## Results

**Figure 1:**
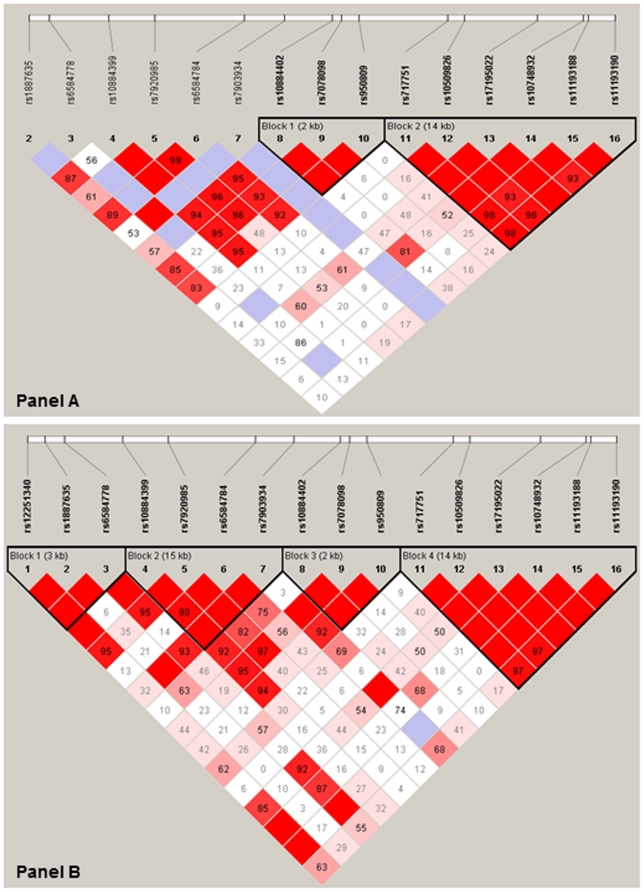
LD patterns of SNPs rs10884402, rs7078098 and rs950809. a) NIA-LOAD dataset (controls). b) Caribbean Hispanic dataset (controls).

Table 1 shows the characteristics of the study populations. In the NIA-LOAD dataset, 11 SNPs were significantly associated with the memory savings score after correction for multiple testing (rs10491052, rs11192998, rs7091546, rs10509823, rs1887635, rs6584784, rs7078098, rs950809, rs596577, rs7083707, rs7922128; 0.006<p<0.04) and in the Caribbean Hispanic dataset two SNPs were associated (rs10884402 (p = 0.007) and rs2149196 (p = 0.04)). rs10884402 (A allele associated with lower scores in the Caribbean Hispanics, table 2), and rs7078098 and rs950809 (C alleles associated with higher scores in the NIALOD dataset) constitute a block of three adjacent SNPs that are 2.4 kb apart and are in LD in both datasets (figures 1a and 1b). In a meta-analysis of both datasets, all three SNPs were significantly associated with the savings score (table 2): corresponding to the separate analyses of both datasets, the A allele of rs10884402 was associated with lower scores, while the C alleles of rs7078098 and rs950809 were associated with higher scores. When the analyses were stratified by AD status, the associations of all 3 SNPs was driven by cases and not present in the controls (table 2). When the analyses were stratified by APOEe4 carrier status, the associations were similar in both APOE groups.

**Table 2:** Single marker associations of SNPs rs10884402, rs7078098 and rs950809 with the memory savings score.

						NIA-LOAD			Caribbean Hispanics			Meta-analysis	
	SNP name	bp	Role	Alleles	minor allele	β	SE	P	β	SE	P	β	p
**ALL**	rs10884402	108,782,932	Intron 1	A/G	A	−0.10	0.07	0.129	−0.15	0.06	0.008	−0.13	0.003
	rs7078098	108,783,778	Intron 1	C/T	C	0.18	0.07	0.007	0.03	0.05	0.623	0.09	0.035
	rs950809	108,785,365	Intron 1	C/T	C	0.17	0.06	0.008	0.09	0.05	0.075	0.12	0.002
**Controls**	rs10884402	108,782,932	Intron 1	A/G	A	−0.08	0.05	0.118	0.03	0.06	0.580	−0.03	0.419
	rs7078098	108,783,778	Intron 1	C/T	C	0.09	0.05	0.092	−0.03	0.06	0.562	0.03	0.407
	rs950809	108,785,365	Intron 1	C/T	C	0.09	0.05	0.090	−0.02	0.06	0.681	0.04	0.326
**Cases**	rs10884402	108,782,932	Intron 1	A/G	A	−0.21	0.28	0.463	−0.15	0.07	0.026	−0.15	0.019
	rs7078098	108,783,778	Intron 1	C/T	C	0.60	0.26	0.023	0.09	0.07	0.182	0.12	0.061
	rs950809	108,785,365	Intron 1	C/T	C	0.51	0.25	0.041	0.12	0.07	0.067	0.15	0.021

β = beta coefficient, SE = standard error, p = p-value. All models are adjusted for Population Stratification, sex, APOE-ε4 and age-at-onset or age-at-examination. SNPs significant at a 0.05 α-level are underlined. All p-values are corrected for multiple testing using the False Discovery Rate (FDR) [30].

Consistent with the single marker analyses, in the 3-SNP sliding window haplotype analyses the corresponding ATT haplotype for SNPs rs10884402|rs7078098|rs950809 were associated with lower scores in both datasets (table 3). In addition, the complementary GCC haplotype was associated with higher scores in the NIA-LOAD dataset. Again, these associations held up in meta-analyses of both datasets, were driven by cases, and not influenced by APOe4 carrier status.

**Table 3:** Haplotype associations of SNPs rs10884402, rs7078098 and rs950809 with the memory savings score.

	NIALOAD						Caribbean Hispanics					Metaanalysis	
	SNPS	HAPLOTYPE	FREQ	BETA	STAT	P	HAPLOTYPE	FREQ	BETA	STAT	P	BETA	P
**All**	rs10884402|rs7078098|rs950809	GCC	0.36	0.26	2.25	0.02	GCC	0.36	0.05	0.87	0.38	0.09	0.08
	rs10884402|rs7078098|rs950809	GTC	0.11	0.10	0.52	0.60	GTC	0.11	0.19	2.30	0.02	0.17	0.02
	rs10884402|rs7078098|rs950809	ATT	0.35	−0.26	−2.18	0.02	ATT	0.29	−0.19	−3.35	0.0009	−0.2	0.00008
	rs10884402|rs7078098|rs950809	GTT	0.17	−0.09	−0.58	0.56	GTT	0.23	0.06	0.93	0.35	0.04	0.52
**Cases**	rs10884402|rs7078098|rs950809	GCC	0.35	0.57	2.20	0.02	GCC	0.36	0.10	1.52	0.13	0.12	0.04
	rs10884402|rs7078098|rs950809	GTC	0.11	−0.02	−0.04	0.97	GTC	0.09	0.09	0.76	0.44	0.08	0.46
	rs10884402|rs7078098|rs950809	ATT	0.37	−0.24	−0.85	0.39	ATT	0.32	−0.16	−2.40	0.01	−0.16	0.01
	rs10884402|rs7078098|rs950809	GTT	0.17	−0.63	−1.82	0.07	GTT	0.22	0.04	0.48	0.62	0.003	0.96
**Controls**	rs10884402|rs7078098|rs950809	GCC	0.38	0.08	1.56	0.12	GCC	0.36	−0.001	−0.04	0.97	0.03	0.28
	rs10884402|rs7078098|rs950809	GTC	0.10	0.02	0.28	0.77	GTC	0.13	0.01	0.17	0.86	0.01	0.75
	rs10884402|rs7078098|rs950809	ATT	0.33	−0.08	−1.44	0.14	ATT	0.27	−0.002	−0.04	0.96	−0.04	0.29
	rs10884402|rs7078098|rs950809	GTT	0.18	−0.03	−0.41	0.68	GTT	0.24	−0.003	−0.05	0.95	−0.01	0.76

Freq = haplotype frequency, Beta = regression coefficient, Stat = Test statistic (T), p = p-value. All models are adjusted for Population Stratification, sex, APOE-ε4 and age-at-onset or age-at-examination. Haplotypes significant at a 0.05 α-level are underlined.

## Discussion

The findings reported here suggest that genetic variation in *SORCS1* is associated with memory performance. Three intron 1 SNPs (rs10884402, rs7078098 and rs950809) were associated in the NIA-LOAD and Caribbean Hispanic datasets with memory retention in single marker and haplotype analyses. In addition, all three SNPs were significantly associated in a meta-analysis including both datasets. When the analyses were stratified by AD status, these associations were restricted to cases.

Our results are consistent with previous reports that genetic variations in *SORCS1* are associated with AD and could affect APP processing [4], [5], [31]. Memory is the cognitive domain predominantly affected by AD, and is associated with changes in Aβ levels [32], [33], [34]. The three identified SNPs associated with memory retention are in LD and it seems likely that they point to the same disease associated variant. Of note, they are located between 108,782,932–108,785,365 bp in intron 1, and are thus in close genetic distance to the SNPs that were associated with AD in our previous report (located at 108,719,950–108,868,606 bp in intron 1), rs601883 reported by Li et al. [4] (at 108,904,435 bp in intron 1) and rs600879 reported by Grupe et al. (at 108,913,108 bp in intron 1) [31]. The fact that the associations were present only in cases in the stratified analyses suggests that the causative variation(s) identify an endophenotype, cognitive decline, rather that AD per se.

A limitation of this study is that we used only baseline measures of cognition rather than change in cognition over time. However, the principle of Mendelian Randomization in genetic association studies overcomes the issue of reverse causation as the inheritance of genetic variants is independent of -that is randomized with respect to- the inheritance of other traits.

Although the identity of the specific AD and memory associated sequence variations in *SORCS1* remain to be determined, our results support a role for *SORCS1* in AD and suggest that genetic variation in or close to intron 1 in *SORCS1* might affect AD risk and memory performance. Additional studies will be needed to determine whether carriers of alleles associated with differential risk for AD and cognitive performance are indeed protected and that protection arises because of high levels of expression of SorCS1.
